# Beyond Conventional Operations: Embracing the Era of Contemporary Minimally Invasive Cardiac Surgery

**DOI:** 10.3390/jcm12237210

**Published:** 2023-11-21

**Authors:** Lilly Ilcheva, Petar Risteski, Igor Tudorache, Achim Häussler, Nestoras Papadopoulos, Dragan Odavic, Hector Rodriguez Cetina Biefer, Omer Dzemali

**Affiliations:** 1 Department of Cardiac Surgery, University Hospital Zurich, 8091 Zurich, Switzerland; lilly.ilcheva@usz.ch (L.I.); petar.risteski@usz.ch (P.R.); igor.tudorache@usz.ch (I.T.); achim.haeussler@usz.ch (A.H.); nestoras.papadopoulos@stadtspital.ch (N.P.); dragan.odavic@stadtspital.ch (D.O.); hector.rodriguez@usz.ch (H.R.C.B.); 2 Department of Cardiac Surgery, Zurich City Hospital—Triemli, 8055 Zurich, Switzerland

**Keywords:** cardiac surgery, minimally invasive cardiac surgery, innovative tools, historical development, prospects, technological advancements

## Abstract

Over the past two decades, minimally invasive cardiac surgery (MICS) has gained a significant place due to the emergence of innovative tools and improvements in surgical techniques, offering comparable efficacy and safety to traditional surgical methods. This review provides an overview of the history of MICS, its current state, and its prospects and highlights its advantages and limitations. Additionally, we highlight the growing trends and potential pathways for the expansion of MICS, underscoring the crucial role of technological advancements in shaping the future of this field. Recognizing the challenges, we strive to pave the way for further breakthroughs in minimally invasive cardiac procedures.

## 1. Introduction

Over the last two decades, there has been a significant shift from traditional to minimal invasive cardiac surgery (MICS), driven by rapid technological advancements [1,2,3,4,5,6,7,8]. In 2021, Germany reported 36.8% of the performed aortic valve (AV) surgeries and 55.7% of all mitral valve (MV) surgeries to be in minimally invasive technique [9]. Moreover, an increase in the number of European institutions performing robotic cardiac surgery, growing from 13 in 2016 to 26 centers by 2019, has also been observed [4]. In our institution, 75% of all cardiac surgeries are minimally invasive, and all staff surgeons are trained to perform the procedures in this manuscript. The growing adoption of MICS can be attributed to two primary factors. Firstly, it responds to the global imperative to combat cardiovascular diseases. Secondly, it is driven by acknowledging the myriad benefits of minimal access techniques in cardiac surgery [10]. These techniques encompass reduced surgical trauma, decreased postoperative pain, shorter hospitalization duration and costs, lower infection risk, faster recovery, quicker resumption of routine activities, and improved cosmetic outcomes [6,7,11,12,13,14,15,16]. MICS is defined by the Society of Thoracic Surgeons (STS) by two criteria: first, the use of smaller incisions and deviation from the conventional median sternotomy (MS), and second, performing surgery without cardiopulmonary bypass (CPB) [17,18]. The reduced invasiveness has been associated with reduced systemic inflammation, blood transfusion requirements, renal dysfunction, and vascular and neurological complications and shorter cross-clamp time [11,12,14,15,16,19,20,21,22,23,24]. Although MICS is technically more demanding and initial reports have demonstrated longer cross-clamp times in the MICS group, we have observed a decrease in cross-clamp timing, especially in minimally invasive mitral valve surgery (MIMVS), as shown by data published by the authors [25].

The field of cardiac surgery has been equipped with tools, such as video-assisted thoracoscopic and robotic technology, as well as advancements in perfusion techniques and transesophageal echocardiography, enabling the progression towards less invasive procedures.

For this review, MICS will be defined as partial upper or lower mini-sternotomy and left or right thoracotomy, providing avenues for the treatment of valvular heart disease (VHD), coronary artery disease (CAD), and aortic pathology; excision of left atrial tumors; and atrial septal defect (ASD) repair, along with the deployment of mechanical circulatory support (MCS) devices through less intrusive techniques [12,15,26,27,28,29]. Furthermore, advancements in port-access cardiac surgery and robotic-assisted cardiac operations in valve and coronary surgery will be used as part of MICS and have been proven to be viable alternatives to MS [5,6,30,31,32,33,34].

The current work aims to provide an overview of the history of MICS, its current state, and its future prospects and highlights its advantages and limitations.

## 2. Minimally Invasive Cardiac Surgery: Development and Current Standard

### 2.1. Minimally Invasive Coronary Revascularization

#### 2.1.1. Coronary Artery Bypass Grafting (CABG)

After performing the first successful open-heart surgery on CPB in 1953, MS became the gold standard incision in cardiac surgery due to its safety, durability, and ease of reproducibility [35]. The landscape of conventional cardiac surgery began to change in 1967 with Kolesov’s groundbreaking procedure of performing coronary artery bypass grafting (CABG) on the beating heart via a left thoracotomy [36]. In the early 1990s, surgeons such as Benetti, Calafiore, Subramanian, and Boonstra conducted the first series of minimally invasive direct coronary artery bypasses (MIDCABs) in cases of left anterior descending artery (LAD) stenosis through a lateral thoracotomy [37,38,39,40]. They demonstrated the durability and safety of minimally invasive CABG in patients with LAD stenosis (0–3.8% in-hospital mortality, postoperative graft patency rate of 92% to 100%, 93% freedom from cardiac events in the first 30 days, and 92.2% after mean follow-up of 5.6 months) [37,38,39,40]. A significant challenge at the beginning of minimally invasive CABG surgery was accessing the coronary arteries and performing precise anastomoses through a minimal incision. Specialized retractors and stabilizers were developed to overcome the challenges faced during the surgical procedure. These improvements provided better visualization of the internal mammary arteries and the ascending aorta. A cardiac apical positioner was also implemented to manipulate the heart and enhance exposure to the coronary territories. Additionally, an epicardial stabilizer was used to stabilize the graft-to-coronary anastomosis. The anesthesiology team was vital in managing intrathoracic and intracardiac pressure to ensure optimal outcomes.

Technical advancements in the 1990s introduced port-access techniques, video-assisted thoracoscopic surgery (VATS), and non-robotic and robotic endoscopic coronary artery bypass grafting (TECAB) for graft harvesting and anastomosis in minimally invasive CABG [3,8,20,41]. In particular, robotic techniques for harvesting the left internal mammary artery (LIMA) with open graft anastomosis through lateral thoracotomy or completely robotic-performed surgery on a beating or arrested heart emerged as an option for performing CABG in a less invasive method [8,20,30]. While the initial application of TECAB focused on revascularizing left coronary vessels, it has been expanded to include total endoscopic harvesting of the right internal mammary artery (RIMA) and revascularization of the right coronary system [41]. Additionally, instances of TECAB using bilateral internal mammary arteries have yielded promising results [42].

A literature review by Bonatti et al. of minimally invasive CABG in the last 25 years reported six different types of minimally invasive surgical revascularization [6].

MIDCAB under direct vision was the most common minimally invasive coronary revascularization method, with 46.9% of all analyzed minimally invasive CABGs [6]. Authors reported a 1.6% conversion rate to MS, 1.3% wound infections, and a 5-year survival rate of 91% in this group [6]. In their prospective two-center study of patients undergoing MIDCAB, McGinn et al. reported complete revascularization in 95% of cases, and the perioperative mortality rate stood at 1.3%, with a 3.8% rate of conversion to MS and 7.6% of patients requiring CPB [43]. The average hospital stay was six days [43]. During the mean follow-up of 19.2 ± 9.4 months, 3% of patients needed further percutaneous revascularization [43]. A case-matched study by Lapierre et al. demonstrated that MIDCAB patients had a statistically significant shorter median hospital stay (5 days vs. 6 days) and a faster median time to return to total physical activity (12 days vs. 36 days) compared to off-pump coronary artery bypass (OPCAB) [44]. In addition, Ziankou et al. indicated that patients undergoing minimally invasive CABG had shorter hospital stays (4.5 days vs. 7.5 days) and a decreased median time to resume complete activities by four times (14 days vs. 56 days) when compared to those undergoing CABG through MS [45].

Video-assisted CABG is a combination of thoracoscopic harvesting of the internal mammary artery (IMA) and direct coronary bypass grafting through a mini-thoracotomy without cardiopulmonary bypass is another method for minimally invasive coronary revascularization without cardiopulmonary [46]. Video-assisted CABG allows the harvesting of the IMA at full length and enables a direct lateral view of the graft rather than the divergent view that the surgeon faces in MIDCAB. The initial results by Benetti et al. demonstrated the absence of mortality and myocardial infarction (MI) and 0% morbidity during the hospital stay [46]. Antona et al. reported a 2.4% rate of acute MI and a 95.2% graft patency rate in the first month preoperatively. During the mean follow-up of 8.7 months, no deaths were registered, and there was no recurrence of angina pectoris symptoms [47]. The review of Bonatti et al. on video-assisted CABG reported a conversion rate to MS of 4.5%, a 1.4% rate of reoperation due to postoperative bleeding, a 0.4% rate of stroke, a postoperative dialysis rate of 1.3%, a wound infection rate of 1.7%, an in-hospital mortality rate of 0.8%, and a 92% survival rate at five years for the cases analyzed in [6].

TECAB with or without robotic assistance is the second (15.8%) and third (15.5%) frequent procedure for minimally invasive CABG, according to the analysis of Bonatti et al. for the last 25 years [6]. The literature review of Göbölös et al. on robotic TECAB for the past two decades reported conversion rates between 23.1% and 33% in the mid-2000s, which were significantly reduced below 10% in the last ten years [5]. The perioperative mortality rate reported was 0.8%, and there was a 2% incidence of surgical revisions required for postoperative bleeding [5]. Additionally, the incidence of stroke was 1.0%, acute renal failure occurred in 1.6% of cases, and in 13.3% of patients, new postoperative AF was documented [5]. The average duration of hospital stay was 5.8 days [5]. Even though TECAB is considered the most challenging among all minimally invasive CABG procedures due to its high technological requirements, from the standpoint of surgical invasiveness, it is the procedure that causes the most minor tissue damage [5,6]. The considerable investment in robotic equipment, the extensive training for surgeons and their teams, the heavy reliance on complex technology, and the critical interdependence of the surgical team members continue to be subjects of robust debate [6].

#### 2.1.2. Hybrid Coronary Revascularization (HCR)

The combined guidelines from the American Cardiac Societies on percutaneous coronary intervention (PCI) and CABG define hybrid coronary revascularization (HCR) as a procedural strategy that combines the placement of a LIMA graft to the LAD artery with PCI on at least one additional non-LAD coronary artery [48]. Current guidelines from the European Society of Cardiology and the European Association for Cardio-Thoracic Surgery acknowledge HCR with a class IIb recommendation and changed the evidence level from C in 2014 to B in 2018, indicating that it may be considered for certain groups of patients. However, this is suggested to be performed in centers with ample experience in such procedures [49].

Multiple studies have confirmed the long-term higher survival rates and lower rate of major adverse cardiovascular events after CABG vs. PCI in patients with multivessel disease and for treatment of left main stem coronary artery disease, notably surpassing the percutaneous therapy with bare-metal or drug-eluted stents, primarily due to the better patency of the LIMA graft to the LAD [50,51,52,53,54].

Even though the advancements in minimally invasive surgery were reported to result in benefits such as quicker recovery, reduced hospital stays, and potentially fewer complications, surgical revascularization may pose challenges in fragile patients with concurrent health issues [55]. On the other hand, PCI revascularization typically carries a lower risk of immediate complications. Some studies have shown superior outcomes compared to saphenous vein grafts (SVGs) [56]. However, PCI has also been associated with increased intervention rates in patients with multivessel disease and coexisting conditions, such as diabetes mellitus [57,58].

Hybrid coronary revascularization might benefit this particular group of patients by combining the strengths of both methods: minimally invasive surgical revascularization of the LAD and PCI for non-LAD lesions [59]. These procedures are usually staged but may be performed within one treatment session. Extensive research has been conducted over the past decade to evaluate the procedural efficacy, short-term safety, and performance of HCR. The interim results showcased in the review by Moreno and DeRose indicate a graft patency rate for the LIMA-LAD bypass ranging between 93% and 100%, along with a survival rate of 93% after five years [60,61].

The concept of the HCR relies on the possibility of providing a personalized approach to coronary revascularization by combining the minimally invasive techniques of LIMA-LAD anastomosis with the targeted approach of PCI for non-LAD lesions. HCR offers the potential for improved outcomes and reduced risks, especially in high-risk patients [62,63]. However, it is essential to note that HCR is still relatively new compared to well-established conventional procedures, and there is a lack of large randomized controlled trials (RCTs) on this topic. The variety of current studies differ in their chosen surgical and interventional methods, patient selection criteria, approaches to antiplatelet therapy, and one-stop vs. staged approaches. While further research is needed to evaluate its long-term benefits fully, the interim results indicate positive outcomes regarding graft patency and survival rates.

### 2.2. Minimally Invasive Valve Surgery (MIVS)

Valve surgery has experienced a significant evolution within the domain of cardiac surgery due to the extensive adoption of minimal access techniques [16,21]. The initial reports on minimally invasive valve repair and replacement techniques came from prominent figures such as Cohn, Cosgrove, Carpentier, Chitwood, and Mohr during the mid to late 1990s [31,64,65,66,67,68,69]. These techniques encompass a range of approaches, from right lateral thoracotomy (with optional rib resection) and mini- or hemi-sternotomy to more advanced methods like video-assisted repair, port-access procedures, and fully robotic valve surgeries [31,64,65,66,67,68,69]. In aortic or mitral surgery, MICS includes a variety of approaches, utilizing specialized technology, tailored vascular entry for CPB perfusion, improved visualization techniques, reduction in the size of cannulas, and providing increased stability and minimizing intrusion into the surgical field [70,71,72,73]. Venous access for CPB in MICS could be performed through direct central right atrium cannulation and peripheral percutaneous femoral or jugular cannulation with vacuum-assisted venous drainage [73,74]. Arterial access can be achieved through central direct cannulation of the aorta, peripheral axillary, or femoral cannulation, percutaneously or via a small incision [71,75]. The surgeon’s preference often determines the choice of cannulation technique. However, femoral arterial cannulation and the consequent retrograde perfusion have been reported in various studies to be associated with an increased risk of neurological events, especially in patients with preexisting vascular diseases [22,76,77]. In contrast, central cannulation and antegrade perfusion are often associated with a lower risk of cerebrovascular accidents and groin-related complications [71,78].

Regarding femoral cannulation, the trend is toward percutaneous cannulation and arterial closure devices to reduce groin complications such as wound infections and seromas [79,80]. Nonetheless, large RCTs on this topic are still lacking, and there are single-center studies supporting both percutaneous and direct cannulation with groin incision as safe methods with low complication rates [80,81].

Two specific techniques for aortic occlusion and cardiac protection have been utilized: the transthoracic aortic clamp and endo-aortic balloon occlusion [82,83]. A recent analysis of compared outcomes from the STS database for MV surgery between 2017 and 2018 showed that the use of endo-aortic balloon occlusion (EABO) was similar to external aortic occlusion in most significant outcomes, including mortality and the efficacy of mitral valve repair [84]. Additionally, MICS has introduced carbon dioxide in the operative area to reduce the risk of air embolism, effectively decreasing intracardiac air volume and mitigating this potential complication.

Adapting MICS techniques in valve surgery has raised concerns that aiming for smaller incisions may compromise patient safety by reducing the visibility provided by MS with established long-term outcomes. MICS techniques offer superior cosmetic results to minimize invasiveness and surgical trauma, but also, compared to the conventional MS approach, are associated with a low postoperative complication rate and comparable short- and long-term results [16,21].

### 2.3. Minimally Invasive Aortic Valve Surgery (MIAVS)

In the execution of MIAVS, the most commonly employed techniques are the partial upper mini-sternotomy and the right anterolateral thoracotomy approach. On the other hand, the parasternal method and transverse sternotomy are utilized less frequently [85,86,87,88,89,90]. The use of a right upper mini-sternotomy approach, also known as the “J” incision or reversed “L” incision, was pioneered by Cohn et al. in 1997 for aortic valve surgery, followed by the introduction of the “L” incision by Svenson et al. and the reversed “T” partial sternotomy by Gundry et al. [85,89,90]. The partial mini-sternotomy involves a small midline skin incision followed by a deviation of part of the sternum. The partial sternotomy can be made on the patient’s right side (“J” sternotomy), left side (“L” sternotomy), or horizontally (inverted “T” mini-sternotomy) [85,89,90]. Other techniques, such as the lower half T-shaped partial sternotomy, “I” mini-sternotomy, and upper V-type mini-sternotomy, are recognized and implemented by certain surgeons but have lost popularity [91,92,93]. Alongside the evolution of partial mini-sternotomy techniques, various surgical approaches have emerged that do not involve sternal deviation. These include procedures with or without video assistance, such as right anterior or right anterolateral thoracotomy and right infra-axillary thoracotomy [91,92]. Entry into the chest cavity is made through the intercostal space, expanded with either a soft tissue retractor or chest retractor. As previously described, the procedures involve CPB, aortic clamping, and cardioplegia. Special instruments like knot pushers and devices for replacing hand-tied surgical knots have become widely used in MIVS. Multiple studies have substantiated the advantages of MIAVS over conventional MS, including shorter recovery time and hospital stay, reduced blood loss and transfusions, lower infection and AF rates, decreased morbidity and mortality, acceptable cardiopulmonary bypass time, and no differences in neurological outcomes and the quality of myocardial protection [12,26,93,94,95]. Furthermore, MIAVS presents a viable alternative in cases of previous cardiac surgery, providing access with fewer adhesions and, particularly in cases of prior CABG, a safe option to avoid graft injury [96]. A meta-analysis by Chang et al. compared results from MIAVS (through upper mini-sternotomy or right anterior thoracotomy) vs. conventional aortic valve replacement via MS, reporting a lower rate of postoperative atrial fibrillation (0.35 to 0.63; *p* < 0.001) and a shorter hospital stay (0.8 (0.4 to 1.3) days; *p* < 0.01) in the MIAVS group, as well as longer CPB times (12.4 min (range, 5 to 19)) [11]. Similarly, the meta-analysis by Brown et al., analyzing 2054 cases of port-access surgery and 2532 cases of MS, showed additional benefits of shorter ventilation times and less blood loss within 24 h (−2.1 h and −79 mL, respectively) in the minimally invasive cohort [12]. The meta-analysis by El-Andari et al. included 48,606 patients who underwent aortic valve replacement and analyzed the advantages of MIAVS via mini-sternotomy or right anterior thoracotomy over aortic valve surgery through MS [97]. The study reported significantly lower in-hospital and 30-day mortality in the MIAVS group compared to the MS group (*p* = 0.02 and *p* = 0.0006, respectively), reduced rates of renal complications (*p* < 0.00001 MS in comparison to port-access surgery and *p* < 0.0001 MS in comparison to right anterior thoracotomy approach), and fewer wound infections (*p* = 0.02 MS in comparison to port-access surgery and *p* < 0.00001 MS in comparison to right anterior thoracotomy approach) [97]. ICU duration and hospital stay were significantly shorter (*p* = 0.0001 and *p* < 0.0001) in the MIAVS group [97]. Phan et al. analyzed 12,786 patients in RTC and non-RTC studies. They reported reduced perioperative deaths in the MIAVS group compared with the conventional MS arm (1.9% vs. 3.3%), fewer renal failure rates (2.5% vs. 4.2%), lower transfusion incidence (36.0% vs. 52.4%), and shorter intensive care stay (−0.60 days) and hospitalization duration (−0.60 days) in the MIAVR group, with a similar mortality rate compared to the conventional group via MS [13]. El-Sayed Ahmad et al. reported 100 cases of video-assisted MIAVS via right anterior thoracotomy with the absence of intraoperative conversion rate to MS, postoperative cerebrovascular events, rethoracotomy for bleeding, and valve-related reoperation and no cases of death in a 30-day follow-up [98]. Olds et al. compared 503 cases of MS, partial upper sternotomy, and right anterior thoracotomy for AV replacement and demonstrated superior results in shorter bypass times (82 (IQ 67–113) minutes vs. 117 (93.5–139.5) vs. 102.5 (85.5–132.5), *p* <  0.0001), a lower incidence of prolonged ventilator support (3.75% vs. 9.17 and 12.9%, respectively (*p*  =  0.0034)), shorter ICU hospitalization (6 (IQ 5–9) days vs. 7 (5–14.5) vs. 9 (6–15.5), respectively (*p* <  0.05)) [24]. The 30-day mortality was lowest in the thoracotomy group (1.5%), followed by the partial upper sternotomy group (1.67%), and was the highest in the MS group (5.17%) [24]. Bakhtiary et al. analyzed 513 cases of video-assisted anterior thoracotomy and reported a 1.5% rate of cerebrovascular events, 1.4% rate of pacemaker postoperatively, 0.4% of paravalvular leak, 0.2% rate of conversion to MS, rethoracotomy rate of 2.1%, 0.6% rate of wound infections, 0% intraoperative mortality rate, 0.4% 30-day mortality rate, and 1.4% mortality rate for the total follow-up [99]. Similarly, Hussain et al. reported lower rates of renal failure (OR: 0.52; 95% CI 0.37 to 0.73, *p* < 0.001) and new-onset AF (OR: 0.78; 95% CI 0.67 to 0.90, *p* < 0.001) in the MICVS group, as well as reduced prolonged intubation (OR: 0.50; 95% CI 0.29 to 0.87, *p* = 0.01), shorter ICU stay (−0.42; *p* < 0.001), shorter time to discharge (−2.79; *p* < 0.001), and reduced mortality (OR: 0.58; 95% CI 0.38 to 0.87, *p* < 0.01) [100]. The presented study results suggest that MIAVS has slightly superior short-term results over the MS approach for aortic valve surgery. However, it is essential to note that these findings could be significantly skewed by variables such as the bias in patient selection and the surgeon’s enthusiasm, expertise, and reputation, mainly when reporting on novel minimally invasive surgical techniques. Given the absence of solid evidence, there is a need for future prospective RCTs to directly compare the mini-sternotomy and lateral thoracotomy approaches to determine their relative benefits and risks conclusively.

### 2.4. Minimally Invasive Aortic Surgery (MIAS)

Significant progress in minimally invasive aortic root and arch surgery has been made with the emergence of the partial upper sternotomy approach. Upon establishing the safety and feasibility of partial sternotomy for aortic valve surgery, further advancements have been made in its application within complex aortic surgical procedures for treating the aortic root, ascending aorta, and aortic arch.

Aortic root surgeries with valve replacement (Bentall procedure), reimplantation (David procedure), or remodeling (Yacoub procedure) are challenging operations due to their technical demands and complexity, as well as the need for an experienced surgeon to perform the procedure.

Nevertheless, Mikus et al. presented a series of 53 patients undergoing Mini-Bentall through partial upper surgery with direct central atrial and venous cannulation [101]. Compared to a selected subgroup of 112 patients undergoing Bentall procedures via MS during the same period, Mini-Bentall showed slight superiority in terms of postoperative outcomes, with shorter operative times, a lower incidence of atrial fibrillation, reduced postoperative ventilation times, and 0% in-hospital mortality [101]. Shah et al. compared in-hospital results and 1- and 3-year mortality for 48 patients who underwent Mini-Bentall and 49 who underwent the Bentall procedure via MS between 2009 and 2019 [23]. The Mini-Bentall group had significantly shorter ventilation times (5.5 h vs. 17 h, *p* < 0.001) and fewer reoperations for bleeding (0% vs. 8.2%, *p* = 0.043) [23]. There were no substantial differences noted in CPB duration (165 min vs. 164 min *p* = 0.619), aortic cross-clamp times (139 min vs. 137 min *p* = 0.948), or lengths of stay in both the intensive care unit and the hospital (6 days vs. 7 days *p* = 0.086) [23]. Zero mortality rates were documented in both groups at 1- and 3-year follow-ups [23]. The review by Sef et al. encompassed various non-randomized observational and comparative studies, highlighting outcomes of the David procedure performed via partial or full sternotomy with central arterial and either central or peripheral venous cannulation [102]. Thirty-day mortality ranged from 0% to 3.3% [102]. Several studies noted reduced requirements for blood products and a relatively shorter ICU stay, averaging between 1.1 and 3 days [102]. Additionally, most studies reported favorable early echocardiographic outcomes, with postoperative aortic insufficiency of grade 1 or less seen in 84.6% to 100% of patients [102].

In another extensive study, Harky et al. analyzed 2765 patients who underwent aortic root surgery in minimally invasive technique or MS across eight comparative studies [14]. Their findings indicated that the minimally invasive approach showed a reduction in CPB time (101.7 ± 33.5 min vs. 109.6 ± 52.9 min, *p* = 0.009), a decrease in blood transfusion rates (1.92 ± 3.17 units vs. 2.75 ± 5.64 units, *p* = 0.01), lower intraoperative mortality (0.411% vs. 1.34%, *p* = 0.02), and shorter stays in intensive care (1.41 ± 1.75 days vs. 2.31 ± 2.28 days, *p* = 0.0009) and the hospital (6.81 ± 3.76 days vs. 7.66 ± 4.41 days, *p* = 0.03) [14]. However, no significant differences were found between the two techniques in aortic cross-clamp time (76.1 ± 24.7 min vs. 78.0 ± 31.5 min, *p* = 0.28), total operation time (252.8 ± 56.3 min vs. 249.7 ± 54.1 min, *p* = 0.31), re-exploration for bleeding, stroke rate, wound infection rate, and duration on mechanical ventilation [14].

Tabata et al. conducted a 5-year follow-up on 128 patients who underwent ascending aortic, aortic arch, and root surgery via upper mini-sternotomy. They compared the results to those of a matched cohort group who underwent aortic operations through MS [103]. The study reported a shorter median length of stay (5 vs. 6 days, *p* = 0.020) and fewer units of red blood cell transfusion (2 vs. 2.5 units, *p* = 0.020) for the minimally invasive group [103]. The 5-year survival rate was 97.2%, with no significant difference between the two groups [103]. Moreover, Svensson et al. conducted a series of studies focusing on minimally invasive ascending aorta surgery, including cases of reoperations [85,104,105]. They reported a shorter postoperative hospital stay for the MIAS group (6.2 vs. 8.2 days; *p* = 0.0055), less postoperative pain, reduced use of intravenous narcotics (morphine 20.6 mg vs. 40.9 mg; *p* = 0.0028), and earlier discharge (5.1 vs. 8.1 days; *p* < 0.0001) [85,104,105]. They also noted low levels of postoperative stroke (0–3.7%), an absence of reoperations, and a 30-day survival rate of 98.5–100% [85,104,105]. In a meta-analysis conducted by Rayner et al., comparing surgery on the ascending aorta and root through both MS and minimally invasive approaches, it was observed that patients undergoing MS experienced extended hospital stays (*p* < 0.001) and prolonged durations in the ICU (*p* < 0.001) [106]. MS patients were also more likely to require reoperation for bleeding (*p* = 0.024) and were more susceptible to renal impairment (*p* = 0.019) [106]. Mortality, stroke, and renal impairment incidence were similar across both groups [106].

Furthermore, pioneering progress was made in the minimally invasive treatment of extensive aortic pathology involving the aortic arch combined with hybrid procedures on the descending aorta via partial upper sternotomy. In a single-center series performing minimally invasive complex aortic procedures, aortic arch repair via upper mini-sternotomy was reported as a safe and effective surgical method, with early and mid-term outcomes comparable to the results obtained with conventional sternotomy [98,107]. Risteski et al. reported that among 123 consecutive patients who underwent aortic arch repair through partial mini-thoracotomy, there was a zero rate of conversion to full sternotomy and an early mortality rate of 3.3%, with permanent and temporary neurological deficits in 4.9% and 8.1% of cases, respectively [107]. Among those who underwent frozen elephant trunk repair (33% of the cohort), the rate of spinal cord injury was 3.3% [107]. After five years, the survival rate was estimated at 80%, and the freedom from reoperation was 96% [107]. Two smaller series on this topic were published by Iba et al. (22 patients) and Goebel et al. (21 patients) [108,109]. Iba et al. reported no early deaths, permanent neurological deficits, or spinal cord injuries; a 5% intraoperative conversion rate to full sternotomy due to bleeding; and a 14% rate of re-exploration due to bleeding [108]. Following this line, Goebel et al. reported no conversions to MS during the initial surgery, a 9.5% rate of rethoracotomy due to bleeding, no permanent strokes, and in-hospital mortality of 4.8% [109]. Even though minimally invasive aortic surgery still lacks a large study series and long-term follow-up, the existing studies suggest that MIAS could be performed safely and with superior early results compared to aortic surgery via MS.

### 2.5. Minimally Invasive Mitral Valve Surgery (MIMVS)

The first series of MIMVS cases was published by Mohr and Chitwood in the late 1990s, followed by Carpentier, who performed the first video-assisted MIMVS in 1996 [31,66,69]. Currently, the most common approach for MIMVS is through a right anterolateral thoracotomy or partial upper mini-sternotomy, under direct vision or with video assistance. In a meta-analysis, Sündermann et al. demonstrated the benefits of MIMVS compared to conventional MS in the following aspects: shorter ICU stay (44 ± 30 h in MIMVS vs. 66 ± 47 h in MS group, *p* < 0.001); reduced dependence on respirators (12.3 ± 11.2 h in MIMVS vs. 22.3 ± 29.1 h MS group, *p* = 0.001); shorter hospital stay (7.6 ± 3.2 days vs. 9.4 ± 3.4); decreased blood volume in drainages (674 ± 288 mL in MIMVS vs. 775 ± 292 mL in MS group, *p* < 0.001); fewer blood transfusions (37% in MIMVS vs. 45% in MS group, *p* = 0.004); and non-significant differences in the rates of rethoracotomy (3.8% in MIMVS vs. 3.2 in MS, *p* = 0.13), stroke (1.7% in the MIMVS group vs. 1.6% in the CS group), new AF (25% in MIMVS and 29% in MS, *p* = 0.07), and 30-day all-cause mortality (1.4% in MIMVS vs. 1.7% in MS) [15]. Even though longer CPB time (MIMVS 142.6 ± 26.5 vs. 107.7 ± 25.2 in MS group, *p* < 0.001) and longer cross-clamp time (MIMVS 93.7 ± 31.3 vs. 74.2 ± 27.5 in MS group) were documented in the MIMVS group, there was no significant difference in the rate of new renal insufficiency in both groups (2.1% in MIMVS and 2.1% in MS, *p* = 1) [15,100]. A recent meta-analysis by Eqbal et al. evaluated 119 studies, of which 8 were RCTs and 111 were observational [32]. They found that MIMVS was associated with a shorter hospital stay in both observational studies and RCTs (RCT: mean difference (MD): −2.2 days; observational: MD: −2.4 days) [32]. The observational studies also indicated that MIMVS could reduce the need for blood transfusions, with fewer units transfused per patient (MD: −1.2 units) and a lower rate of patients requiring transfusions (relative risk, 0.7) [32]. Furthermore, observational data pointed to a lower mortality rate associated with MIMVS (RR, 0.6; *p* < 0.001) [32]. In contrast, RCTs failed to confirm the results of observational studies [32].

In their meta-analysis of RCTs and case–control studies, Modi et al. reported slightly lower mortality rates for MIMVS, with 1.1% for MV repair and 4.9% for MV replacement, compared to 1.5% and 5.5% for surgery through MS [16]. The meta-analysis conducted by Bonatti et al. reported that at a 5-year follow-up, the rates of patients who remained free from moderate and severe MV regurgitation were 12% and 7.2%, respectively [7]. Moreover, McClure et al. reported a single-center experience with 1000 patients, demonstrating freedom from recurrent severe mitral valve regurgitation at 1, 5, and 10 years to be 99% ± 1%, 87% ± 2%, and 69% ± 4%, respectively [110].

In line with other investigators, Feirer et al. demonstrated extended long-term follow-up results from a single-center experience of 1194 cases of non-robotic MIMVS [111]. The survival rate at 5, 10, and 20 years was 96.7%, 91.6%, and 80.0%, respectively, and the incidence of reoperation was 4.4% at 5 years, 10.3% at 10 years, and 16.7% at 20 years [111].

In 1998, Carpentier et al. achieved the first fully automated MV repair using robotic telemanipulation with the Da Vinci Surgical System [66]. Robotic MIMVS is suitable for both degenerative and functional mitral valve pathologies and simultaneous tricuspid valve repair and ablation procedures [33,112,113,114,115,116]. Murphy et al. reported the results of 1257 robotic mitral valve surgeries [33]. They observed 0.9% intraoperative mortality, 0.7% strokes, and satisfying postoperative results with mild or less mitral valve regurgitation in 98.3% [33]. In 18% of the cases, a concomitant atrial ablation was performed, and 11% of the patients received a concomitant tricuspid valve repair [33]. In the conducted follow-up of 50 ± 26 months, 44 patients (3.8%) required mitral valve reoperation [33]. Suri et al. reported an overall survival of 99.5% at 5 years, 5-year freedom from mitral valve recurrence in 94.6%, and 5-year freedom from reoperation in 97.7% [114,116]

A meta-analysis conducted by William and colleagues indicated survival rates of 99.2% at 1 year, 97.4% at 5 years, and 92.3% at 10 years [117]. Additionally, the freedom from mitral valve reoperation 8 years after robotic-assisted mitral valve repair was 95.0%, and the freedom from moderate or worse mitral valve regurgitation at 7 years stood at 86.0% [117]. The early postoperative complication rate was relatively low, with a 0.2% incidence of all-cause mortality and 1.0% occurrence of cerebrovascular accidents. Reoperations due to bleeding were documented in 2.2% of cases, and the mitral valve repair success rate was 99.8% [117]. Patients spent approximately 22.4 h in the intensive care unit, and the hospital stay had an average duration of about 5.2 days [117].

In summary, MIMVS has become increasingly popular over the conventional sternotomy approach for mitral valve interventions in the last two decades, offering several benefits. Using smaller incisions or less invasive access points allows shorter hospital stays, reduced complications, and improved cosmetic outcomes. The integration of robotic techniques further enhances the effectiveness and safety of MIMVS. With ongoing advancements in this field, MIMVS is poised to continue evolving and playing a significant role in the future of cardiac surgery.

### 2.6. Minimally Invasive Tricuspid Valve Surgery (MITVS)

Tricuspid valve pathology poses a significant challenge regarding prognosis when managed solely through medical interventions. Patients with tricuspid valve pathology often present with concurrent health issues stemming from systemic venous congestion and reduced cardiac output. Similarly to the MIMVS, MITVS could be performed through mini-thoracotomy with or without video assistance for isolated or concomitant tricuspid valve surgery and with robotic assistance simultaneous with MIMVS [118,119]. Although there are limited studies reporting the results of MITVS and even fewer prospective randomized trials comparing it to conventional surgery, the available data indicate significant reductions in mortality, postoperative pain, pacemaker implantation rates, blood loss, and rethoracotomy rates with MITVS, as reported by Abdelbar et al. [120]. Tricuspid valve surgery is rarely performed as an isolated procedure; more often, it is performed as a concomitant surgery. The surgical management of double or triple heart valve pathology exhibits a level of complexity that surpasses that of singular valve interventions. Adopting less invasive modalities may further augment the technical intricacy inherent to these operations [121]. However, recent technological innovations in minimally invasive procedures engender operative conditions that parallel those in traditional surgical approaches. Risteski et al. demonstrated that MICS through partial upper sternotomy could provide access to all heart valves and offers the possibility for triple valve surgery without technical limitations, offering superior results for a lower rate of wound dehiscence and in postoperative bleeding, intensive care unit and hospital stay, and early deaths [121]. Kaimov et al. demonstrated promising results through a limited single-access via lateral right thoracotomy for double and triple valve surgery, with a technically feasible approach in selected patients [122].

As experience accrues, the specific indications for minimally invasive approaches will be further refined, and long-term outcomes of double and multiple heart valve surgeries via mini-thoracotomy will be documented. Nonetheless, despite the burgeoning enthusiasm, the importance of caution cannot be overstated, given that traditional cardiac operations continue to demonstrate proven long-term success alongside perpetually diminishing morbidity and mortality rates, thus remaining as our benchmark for comparative evaluation.

### 2.7. Minimally Invasive Surgery for Atrial Fibrillation (AF)

Atrial fibrillation (AF) is the most prevalent sustained arrhythmia, posing a significant risk of thromboembolic events and potentially leading to life-threatening conditions. Surgical intervention for AF is commonly performed alongside other cardiac surgeries in symptomatic individuals or asymptomatic patients with minimal additional risk.

Primary cardiac surgery becomes a consideration when AF persists despite medical interventions or when patients have experienced unsuccessful attempts at catheter ablation or have contraindications to such procedures. The initial Maze procedure, developed in 1987, has been regarded as the gold standard for surgical ablation of AF [123,124]. Over time, the Cox Maze III (CM III) procedure emerged as the benchmark for surgical AF treatment in the 1990s [124]. This procedure, performed through MS, involves intricate cut-and-sew lesions in both atria. Despite its effectiveness, the complexity and technical challenges associated with CM III have limited its widespread adoption.

In recent years, the field has witnessed the advancement of the Cox Maze IV (CM IV) procedure, which has demonstrated notable reductions in operative and cross-clamp times and complexity compared to CM III [125]. This progress can be attributed to various innovations, including the development of bipolar or monopolar radiofrequency lamps, cryothermal ablation devices, and refinements in procedural techniques. These advancements have not only reduced invasiveness but also enabled the performance of minimally invasive procedures through video-assisted right mini-thoracotomy [125,126,127]. Almousa et al. reported freedom from atrial tachyarrhythmia and antiarrhythmic drugs at 9, 12, 18, 24, 36, and 48 months postoperatively to be 97.0%, 96.7%, 98.1%, 97.1%, and 100%, respectively after analyzing the data of 135 patients who underwent isolated or concomitant robotic biatrial CM IV [128]. In addition, Ad. et al. reported low perioperative morbidity: the absence of stroke, vascular complications, and conversion to MS and a 5% rate of new pacemaker implantation [127]. Sinus rhythm in a follow-up of 1, 2, 3, 4, and 5 years postoperatively was documented in 93%, 93%, 91%, 91%, and 90% of the cases, and freedom from atrial tachyarrhythmia and antiarrhythmic drugs was 88%, 82%, 76%, 74%, and 73%, respectively, at 1, 2, 3, 4, and 5 years after the CM III/IV [127]. In a systematic review by Je et al., minimally invasive stand-alone surgical procedures for restoring sinus rhythm demonstrated success rates ranging from 70% to 93% after one year, depending on the specific ablation technique [129].

These advancements highlight the evolving landscape of surgical approaches to treating AF, with minimally invasive techniques offering promising results.

In conclusion, the field of surgical treatment for AF has witnessed significant advancements, particularly with the introduction of the CM IV procedure. These advancements have reduced operative and cross-clamp times, improved outcomes, and reduced morbidity. Minimally invasive techniques, such as the video-assisted right mini-thoracotomy, have shown great potential to achieve freedom from AF and improve patient outcomes. The continuous evolution of surgical approaches and the adoption of minimally invasive techniques hold promise for the future of AF treatment.

### 2.8. Minimally Invasive Ventricular Assist Device Implantation

The application of mechanical circulatory assistance, specifically left ventricular assist devices (LVADs), has undergone remarkable growth in managing congestive heart failure. This expansion has outpaced the global number of heart transplants annually. Modern LVADs differ significantly from earlier iterations with their advanced technology and compact design. These advancements have opened the door to minimally invasive surgical procedures for LVAD implantation.

The initial attempt at less invasive LVAD procedures was documented by Pasic et al. in 1999, involving a successful placement through a left anterolateral thoracotomy [130]. Subsequent methodologies were developed to adapt to the large dimensions of first-generation LVADs and newer models like the Jarvik 2000 Heart [131,132].

Contemporary techniques typically require accessing only two thoracic sites: the left ventricular apex and the ascending aorta. Various minimally invasive approaches involve separate incisions, such as a J-shaped upper mini-sternotomy, a right-sided thoracotomy for the aorta, and a left-sided thoracotomy for the apex, allowing two surgeons to work simultaneously, thereby shortening the procedure’s duration [28,131,132,133,134,135]. Minimally invasive LVAD implantation can be performed with or without CPB. Utilizing CPB can provide advantages such as preventing hemodynamic instability and facilitating examination of the left ventricle during the coring process, which can help avoid complications like stroke and pump thrombosis [28]. According to some investigators, LVAD implantation without CPB could reduce hemodilution, systemic inflammatory response syndrome, the adverse effects of pulmonary hypertension, and postoperative RV dysfunction [132] [133,136]. Even though off-pump LVAD implantation may have its benefits, it has potential risks such as severe blood loss and hemodynamic instability [132,133]. Minimally invasive implantation has gained popularity with the reduction in LVAD pump size. Many medical facilities have adopted this approach, noticing advantages such as reduced bleeding, severe right heart failure, blood product transfusion, mechanical ventilation time, and respiratory failure risks. A meta-analysis by Zhang et al. showed no significant differences between minimally invasive and conventional techniques in short- and mid-term mortality, neurological events, and pump thrombosis [134].

Additionally, for patients bridging to transplantation, minimally invasive methods may reduce risks associated with future sternotomy [137]. Despite these advancements, minimally invasive LVAD surgery remains relatively new compared to established cardiothoracic procedures like coronary revascularization or valve surgery. There is a lack of comparative studies that could provide insights into medium-term or long-term outcomes. More comprehensive research will be vital as this technique evolves to further understand its potential benefits and limitations.

## 3. Emerging Technologies in Minimally Invasive Cardiac Procedures

These emerging technologies highlight continuous innovation in minimally invasive cardiac procedures, promising to reduce risks further, enhance patient outcomes, and make these advanced treatments more accessible to a broader patient population. Ongoing research and collaboration between engineers, medical professionals, and industry are essential to realize this potential fully.

### 3.1. Robotic Cardiac Surgery

Integrating robotic-assisted techniques has revolutionized surgical procedures by empowering surgeons with enhanced vision, precision, control, and skill [3]. Robotic systems, such as the Da Vinci XI Surgical System, offer improved visualization, precision, and control for surgeons, resulting in enhanced patient outcomes [3]. The advanced features of the Da Vinci system, such as laser targeting, pre-programmed arrangements, and a refined clutching mechanism, have revolutionized surgical procedures by providing surgeons with an optimal visual field and precise movements [138]. The exceptional 3D visualization offered by the Da Vinci system compensates for the absence of tactile feedback in robotic surgery, allowing surgeons to observe tissue displacement accurately.

One of the significant advantages of robotic instruments is their ability to scale natural movements into precise movements, ensuring smoother and more accurate articulation of devices at the surgical site [139,140]. With its wrist-like movement capabilities, the robotic instrumentation provides surgeons with seven degrees of freedom, surpassing the limitations of traditional minimally invasive surgery instruments [141,142]. These technical benefits have expanded the possibilities of cardiac surgery, allowing for more complex procedures and overcoming the limitations associated with conventional endoscopic devices.

The domain of robotic technology is experiencing a substantial expansion within the sphere of medical equipment. Integrating automated systems within medical apparatuses is perpetually drawing noteworthy interest and investment, signifying a transformative juncture in the healthcare sector. A notable project in this realm, pending approval from the Food and Drug Administration (FDA), is spearheaded by Vicarious Surgical. This system epitomizes an impending era in robotics engineering, striving to foster revolutionary technology with the aspiration of amplifying the efficiency of surgical operations. Access and visualization are gained via a solitary 1.5 cm port [143]. A camera and two robotic instruments can be introduced through this port, thereby maximizing the visualization, precision, and control of instruments for the surgeon operating the device [143].

Further advancement within robotic technologies is the employment of soft robotics in MICS. Soft robotics in cardiac surgery presents a novel avenue for enhancing surgical procedures while minimizing patient trauma. Due to their compliant materials and adaptability, soft robotic systems can proficiently navigate the intricate and delicate cardiac environment [144]. Their capacity to conform to the surroundings allows for precise manipulation with reduced risks, which is essential in cardiac operations. By employing soft robotics, surgeons can achieve improved operational efficacy, better patient outcomes, and reduced recovery times in cardiac surgeries, aligning with the overarching goal of advancing patient-centric medical interventions [144].

Incorporating robotic devices within the MICS field significantly augments surgical procedures’ precision and efficacy. These advanced technological entities aim to minimize surgical invasiveness by facilitating meticulous manipulation within the cardiac arena, potentially reducing patient recovery times and improving overall surgical outcomes [3,5,6,7,20,139,144,145,146]. Nonetheless, robotic surgery remains in its nascent utilization stages, with availability and manageability confined to a limited number of cardiac centers. The advancements in robotics indubitably harbor the potential to broaden the scope and capabilities of contemporary cardiac care.

### 3.2. Virtual Reality (VR) Technology

In the realm of MICS, the advent of new imaging techniques has significantly improved our ability to diagnose, strategize, and perform intricate cardiac procedures, advancing both accuracy and safety. Recognizing both conventional and aberrant anatomical structures before surgery has become essential, especially in minimally invasive procedures or cases requiring reoperation, where a comprehensive visual examination of the anatomical components may not be possible.

Traditional imaging modalities, such as computed tomography (CT) and echocardiography, remain vital for preoperative patient selection and surgical preparation. Nevertheless, contemporary research has expanded into novel imaging methodologies, incorporating augmented reality (AR) and VR for enhanced medical image visualization and real-time operative guidance. Sadeghi et al. demonstrated in a small patient series (6 patients) that VR can assist in predicting the precise size of the required left atrial appendage (LAA) clip by preoperatively measuring the LAA bases [147]. Additionally, VR can be valuable in visualizing anatomical structures, particularly in cases of reoperations, to prevent unintentional injury. It can also aid in optimizing cannulation techniques during surgery by visualizing patients’ vascular structures preoperatively. Furthermore, it can offer advice on the placement of minimally invasive incisions, enhancing the overall surgical approach. AR and VR are now used in cardiac surgery, particularly within MICS, with proven efficacy in pediatric, thoracic, neurosurgical, and urological applications [148,149,150,151]. AR and VR could be beneficial in determining the most suitable surgical approach (conventional vs. minimally invasive), identifying patients not ideal for a minimally invasive procedure, and recommending optimal strategies for valve repair, including ring and valve sizing and types [145,150,151,152].

For procedures such as MIVS, these technologies could offer valuable information and determine the location of the surgical incision, port placement, and visualization of the LIMA and coronary anatomy [147,152]. In the field of mitral valve surgery, VR is tried to be applied to optimize surgical technique, approach, and plan for a particular patient’s mitral valve pathology and postoperative outcomes [153,154,155]. Choi et al. employed VR to generate a patient’s mitral valve from 3D transesophageal echocardiography data to assess the improvement in mitral valve function after ring annuloplasty [155]. Their findings highlighted that virtual mitral valve models allow for a comprehensive assessment of physiological and biomechanical aspects before and after annuloplasty, providing detailed insights into mitral valve function. Al-Maisary and colleagues, using similar simulation models, assessed the degree of displacement of the mitral annulus [153]. Their study showed that different types of annuloplasty rings used in patients with varying annular shapes and dimensions did not result in any significant alterations in the configuration of the mitral annuli following the virtual implantation of the tested annuloplasty rings [153]. Rausch et al. performed a virtual simulation of mitral valve annulus downsizing with ring annuloplasty and demonstrated implantation of an undersized ring had a more significant effect on tissue deformation in the myocardium and mitral annulus [154]. Utilizing such modeling techniques allows surgeons to simulate various levels of annular downsizing in a virtual environment. This will enable them to assess and estimate the impact on mitral valve mechanics before the surgical procedure, aiding in preoperative planning and decision making [154].

These models offer the potential for tailoring the selection of annuloplasty rings to individual patients. They enable personalized virtual assessments of mitral valve biomechanics and function before and after annuloplasty, potentially leading to more precise and effective improvements in mitral valve function.

A well-recognized challenge in MICS is its steep learning curve, resulting in limited adoption and extended procedural duration compared to conventional approaches. Integrating VR principles could represent a critical advancement in this context. By promoting the education and training of aspiring surgeons and facilitating the practice of novel surgical techniques, VR and AR may reduce operating time within MICS, potentially accelerating its adoption and increasing its efficiency [147]. The emergence of new imaging techniques in MICS has undoubtedly enhanced our ability to diagnose, plan, and execute complex cardiac procedures with increased precision and safety.

The application of VR in cardiac surgery is a relatively recent technological development. There have been only a limited number of reports on this subject, with most of them being small-scale studies conducted at single centers. While the findings presented are promising, it is crucial to emphasize the need for more rigorous research methods to assess the feasibility, safety, and validity of the claimed clinical applications of VR in cardiac surgery. More extensive and comprehensive studies are required to provide a more definitive assessment of VR in this field.

## 4. Disadvantages of Minimally Invasive Cardiac Procedures

Minimally invasive cardiac procedures have transformed the field of cardiac medicine, offering treatment options for adult and congenital surgical cases. These procedures are performed through a smaller skin incision, often without or with only partial deviation of the sternum, leading to reduced trauma.

Despite the advantages mentioned above of MICS, this approach has some limitations and challenges. According to the meta-analysis by Dieberg, MICS requires longer cross-clamp time (MD 6.7 min (95% CI 1.24 to 12.17, *p* = 0.02)), longer CPB time (MD 26.68 min (95% CI 10.31 to 43.05, *p* = 0.001)), and longer operation time (MD 55.03 min (95% CI 22.76 to 87.31, *p* = 0.0008)) [25]. Doenst et al. reported longer associations between cross-clamp time and mortality, low cardiac output syndrome, and acute kidney injury (all *p*  <  0.001) [156]. Complications can arise from peripheral cannulation, including increased stroke rates, groin seroma, infections, and, in rare cases, arterial trauma or retrograde aortic dissection [112,157]; moreover, unilateral pulmonary edema (UPE) is an underreported issue in MICS that may increase mortality rates [112,158,159].

In contrast, Lamelas et al. analyzed 2400 patients who underwent MICS via femoral cannulation and demonstrated a 1.17% rate of cerebrovascular events postoperatively, the absence of aortic dissections, a 0.8% rate of compartment syndromes, a 0.7% rate of femoral arterial pseudoaneurysms, and 174 (6.65%) groin wound seromas [157].

Adopting MICS techniques requires extensive training, potentially impacting patient outcomes during the initial adoption phases, as indicated by Vo et al. [160]. As previously outlined, applying MICS necessitates substantial preoperative planning, acquired proficiency, and utilizing specialized instruments and tools. Despite these requirements, there are instances where conversion to MS is unavoidable. Yadava et al. found that this conversion occurs in 2% to 3% of cases, driven by unforeseen complications such as lung adhesions, problems with cannulation, hemorrhage, or atrioventricular rupture [161,162,163]. This transition to MS is not merely a procedural adjustment; it is linked with severe perioperative complications and an elevated 30-day mortality rate that exceeds 23%, particularly in the realm of MIMVS, as reported by Vollrath et al. [163].

## 5. Directions for Future Research

Progressive technological breakthroughs will significantly influence the impending evolution of MICS, enabling further refinement and wider accessibility in cardiac centers, even for patients deemed inoperable. The potential integration of artificial intelligence (AI) and VR aims to enhance the perioperative environment in MICS, provided their safety and efficacy are substantiated. Moreover, harnessing advancements in bioprinting will be pivotal in the realization of in situ tissue repair through tailored bioprinted constructs. Furthermore, persistent investigation to elucidate the comparative merits of MICS against traditional cardiac surgery at molecular and immunological strata will be crucial. Such research could uncover nuanced advantages in diverse fields, such as inflammation management, thereby solidifying the stature of MICS within the dynamic sphere of modern cardiac care.

The continuous innovation in minimally invasive cardiac procedures highlights the emerging technologies that promise to reduce risks further, enhance patient outcomes, and make these advanced treatments more accessible to a broader patient population. Ongoing research and collaboration between engineers, medical professionals, and industry are essential to realize this potential fully.

One of the significant advancements in minimally invasive cardiac procedures is the integration of robotic-assisted techniques. Robotic systems, such as the Da Vinci XI Surgical System, offer improved visualization, precision, and control for surgeons, resulting in enhanced patient outcomes. The advanced features of the Da Vinci system, such as laser targeting, pre-programmed arrangements, and a refined clutching mechanism, have revolutionized surgical procedures by providing surgeons with an optimal visual field and precise movements. The exceptional 3D visualization offered by the Da Vinci system compensates for the absence of tactile feedback in robotic surgery, allowing surgeons to observe tissue displacement accurately.

While current robotic-assisted surgeries require surgeons to be physically present in the operating suite, the future holds the potential for remote telerobotic surgeries [164]. With long-distance telerobotic systems, surgeons can operate from short or long distances, providing tailored medical services to geographically distant or secluded regions. Advancements in robotic manipulation, vision systems, and telecommunications will further enhance the capabilities of telerobotic surgeries in cardiac care. This technological advancement opens doors for expanded applications in cardiac surgery, including thoracic aortic procedures, pediatric cases, and the combination of robotic-assisted techniques with transcatheter valve therapies.

In addition to robotic-assisted techniques, AI has emerged as a transformative technology in surgery [164,165]. AI’s ability to process vast amounts of data, learn patterns, and generate insights is revolutionizing patient care, especially for those with coronary artery disease, heart failure, or rhythm disorders. Recent advancements in AI technologies, including machine learning, natural language processing, artificial neural networks, and computer vision, have led to the development of predictive models and image analysis algorithms. These tools enable clinicians to analyze complex datasets, predict patient outcomes, create personalized treatment plans, and even simulate surgical outcomes. In the planning phase, AI assists surgeons by identifying anatomical variations and suggesting optimal incision strategies. It can also recognize patterns and predict potential surgical complications.

Moreover, from the currently presented data, there is a need for further clinical research to validate and improve the application of MICS, including comparative and RCT studies with traditional cardiac surgery and investigations into molecular and immunological differences.

## 6. Conclusions

The introduction and advancement of MICS represent a significant breakthrough in cardiac surgery. This approach offers numerous advantages, including reduced invasiveness, faster recovery, and improved cosmetic outcomes, all while maintaining comparable results to traditional cardiac surgery techniques. While MICS has yet to be widely embraced, it has successfully navigated various challenges and periods of low acceptance, positioning itself as a promising contender for the future evolution of cardiac surgery. However, to fully realize the potential of MICS, it is crucial to address technical challenges, understand and manage complications, invest in training and education, and prioritize patient suitability. Surgeons and healthcare professionals must undergo extensive training and acquire the necessary skills to perform these procedures effectively. Continuous education and training programs can help enhance proficiency and reduce the learning curve, ultimately promoting wider adoption of MICS. With increased research, randomized controlled trials, and ongoing monitoring, the application of MICS can be validated, refined, and optimized, ultimately shaping the future of cardiac surgery.
